# National Preparedness Month — September 2018

**DOI:** 10.15585/mmwr.mm6735a1

**Published:** 2018-09-07

Each September, CDC, along with 3,000 global, national, regional, and local governments, as well as private and public health institutions, supports emergency preparedness efforts and encourages U.S. residents to take action before, during, and after an emergency. Every community in the United States needs to be ready to respond to an infectious disease outbreak, a chemical or radiologic release, or a natural disaster (*1*). Public health systems need the capacity to scale up and respond to emergencies (*2*).

This year marks the 100th anniversary of the 1918 influenza pandemic, which resulted in an estimated 50 to 100 million deaths (*3*). Planning and preparedness for all types of public health emergencies are vital to keeping communities safe.

This year, CDC is highlighting four areas: 1) personal preparedness, 2) pandemic planning, 3) policy and partnerships, and 4) public health response. Personal preparedness helps communities to be more resilient in the event of an emergency. Through pandemic planning, CDC works to protect the nation from seasonal and pandemic influenza, and through partnerships, CDC plays a pivotal role in state and local readiness. CDC’s Emergency Operations Center and the Division of State and Local Readiness bring together experts and state-of-the-art technology to detect and respond to public health emergencies, such as the recent Zika virus outbreak featured in this issue of *MMWR* (*4*). Additional resources are available at https://www.cdc.gov/phpr/index.htm.
